# Exogenous shocks and the adaptive capacity of family firms: exploring behavioral changes and digital technologies in the COVID‐19 pandemic

**DOI:** 10.1111/radm.12471

**Published:** 2021-03-30

**Authors:** Jonas Soluk, Nadine Kammerlander, Alfredo De Massis

**Affiliations:** ^1^ Institute of Family Business and Mittelstand Entrepreneurship & Innovation Group WHU – Otto Beisheim School of Management Vallendar 56179 Germany; ^2^ Faculty of Economics and Social Sciences University of Bern Bern 3012 Switzerland; ^3^ Centre for Family Business Management Faculty of Economics and Management Free University of Bozen – Bolzano Bozen‐Bolzano 39100 Italy; ^4^ Lancaster University Management School UK; ^5^ International Institute for Management Development Switzerland; ^6^ Institute for Entrepreneurs and Institute for Family Business Zhejiang University China

## Abstract

The COVID‐19 pandemic has been and is currently still affecting organizations of all sizes and in many industries, and research still lacks profound insights into the managerial implications of this phenomenon. In particular, it is unclear how family firms, which are the economic backbone of most of the countries affected by the pandemic, have adapted to COVID‐19. This paper addresses this gap by drawing on a rich body of evidence collected from 90 interviews and secondary data in a longitudinal case study of four German family firms. We develop a framework for understanding how family firms adapt to exogenous shocks such as the COVID‐19 pandemic and find that the exogenous shock further reinforces the family firm’s resource constraints and the family’s fear of losing their socioemotional wealth. These motivational sources, in turn, trigger behavioral changes in both the firm and the family. In addition to a temporarily induced short‐term orientation, these changes manifest in (pseudo)family cohesion, less rigid mental models, and the utilization of digital technologies. Organizational outcomes such as new alliances, digital platforms, and the adaptive capacity of family firms are the result of these behavioral changes. By providing a comprehensive understanding of how COVID‐19 affects family firms, the insights from our study contribute to innovation research, business practice, and policymaking alike. More broadly, we provide innovation scholars with a theoretical comprehension of how exogenous shocks can challenge our canonical understanding of organizations’ (innovative) behavior.

## 1 Introduction

The COVID‐19 pandemic (Li et al., 2020; Zhu et al., 2020) has taken on a global dimension, leading almost all industrialized nations to bring their economies to a shutdown, with massive contact restrictions (Ivanov, 2020) and creating a shortage of both supply and demand in many industries. Such a severe discontinuity in an economic activity constitutes an exogenous shock and has a potentially devastating effect on companies (Kuckertz et al., 2020). Given the restrictions on generating revenues in many industries, firm management is required to take immediate action regarding the safety of employees, and the company’s actual existence can be at stake (Craven et al., 2020). Due to their resource constraints (Feranita et al., 2017; De Massis et al., 2018a), the challenges triggered by the COVID‐19 pandemic particularly apply to private family firms (De Massis and Rondi, 2020), which are the backbone of industrialized economies worldwide (Bornheim, 2000; Shepherd and Zacharakis, 2000). Even in times of crisis (Bjuggren, 2015), family firms not only focus on economic results but also pursue noneconomic goals (Gómez‐Mejía et al., 2007; Berrone et al., 2012), implying that family firms are under particularly high levels of pressure after an exogenous shock such as the COVID‐19 pandemic (Block, 2010; Chirico et al., 2019).

Although entrepreneurship research has studied the role of new venture creation in times of crisis (e.g., Davidsson and Gordon, 2016; Simón‐Moya et al., 2016) and concepts such as “disaster entrepreneurship” (Linnenluecke and McKnight, 2017), we know surprisingly little about how family firms *adapt* to exogenous shocks, that is, how they “manage [themselves] or [their] environment in order to maintain or improve [their] performance, legitimacy, and, hence, survival potential” (Gilbert, 1992, p. 90). This research gap is astonishing because of their idiosyncratic innovation dynamics (König et al., 2013; Duran et al., 2016) – which are particularly driven by the family firms’ tradition and their ability to internalize and reinterpret knowledge (De Massis et al., 2016; Erdogan et al., 2020) – resource scarcity (De Massis et al., 2018a), and entrepreneurial orientation (Zahra et al., 2004) might affect how family firms deal with such crises. To date, research lacks knowledge on how these attributes affect family firms’ adaptation, especially in disruptive situations such as the COVID‐19 pandemic and its aftermath. Although we know that family firms focus on survival due to their transgenerational intentions (Zellweger et al., 2012), we lack knowledge on how they ensure survival in times of crisis that threaten firm survival. This raises the following research questions: (1) *What are the responses of family businesses and business families to exogenous shocks such as the COVID‐19 pandemic?* (2) *How and why do such responses differ among family businesses and business families?*

Addressing those research questions is important for several reasons. First, an explanation of how family firms adapt to exogenous shocks will allow innovation research to understand how family businesses (and business families) cope with crises by introducing innovations. Relatedly, and building on the current scholarly debate on resilience in family businesses, business families, and their linkages (Campopiano et al., 2019), we aim to better understand how such firms can ensure continuity despite an exogenous shock. Due to its extraordinary strength, the COVID‐19 pandemic is a suitable context to explore these questions. Second, more nuanced knowledge about the motivations, behavioral changes, and organizational outcomes of family businesses (and business families) in the pandemic might inform further theorizing on their adaptive capacity and innovation during environmental distress. Third, by acknowledging both the business and the family perspective of family firms, we consider the complexity of these organizations and allow further insights into the interaction of both perspective dimensions before and during crises. To answer the exploratory research questions, we build on a longitudinal multicase study to investigate the adaptation of four family firms to the COVID‐19 pandemic. Our data include 90 interviews with key decision makers in those firms, industry experts, and further secondary data referring to both the period before and after the COVID‐19 outbreak.

We aim to make the following contributions. First, we explain the adaptation and behavioral changes in family firms reacting to exogenous shocks, thereby contributing to management and innovation research in general and family firm literature in particular. More specifically, we reveal how similar behavioral patterns affect family firms’ adaptive capacity, while acknowledging heterogeneity induced by diverse noneconomic goals. Moreover, challenging previous assumptions about family firm idiosyncrasies, we emphasize the necessity to consider exogenous shocks when explaining the adaptability of these firms. Second, we reveal insights for practitioners by explaining how family firms can overcome crises and how they can turn crises into business opportunities (e.g., with digital technologies) (Soluk and Kammerlander, 2021). Third, our insights provide guidance for policymakers and equip them with an in‐depth understanding of the adaptation patterns in family firms, which is a prerequisite to develop support programs and other legislation in a purposeful way. Due to their relevance for economies worldwide, more targeted interventions (for instance, linked to technological premises or behavioral patterns) might be decisive for the economic development of economies in the aftermath of crises such as the COVID‐19 pandemic.

## 2 Research on exogenous shocks, adaptive capacity, and family firms

Exogenous shocks are a central and long‐lasting theme in management research (Hermann, 1963). Although the circumstances surrounding such shocks vary widely (e.g., financial crises or natural disasters), such events have in common that they appear suddenly and entail far‐reaching consequences for involved parties (Doern et al., 2018). More formally, an exogenous shock is defined as “a period of prolonged and widespread crisis in which actors struggle to reconstitute all aspects of social life” (Fligstein and McAdam, 2011, p. 32), while a crisis is defined as a “perception that an individual or set of individuals faces a potentially negative outcome unless some type of corrective action is taken” involving a high degree of “importance, immediacy[,] and uncertainty” (Dutton, 1986, p. 502). Hence, the COVID‐19 pandemic is an exogenous shock, as it has drastic implications for companies that have been affected by contact restrictions and lockdowns in their respective countries (Kuckertz et al., 2020).

One important stream of research has focused on a firm’s *adaptive capacity* when reacting to exogeneous shocks (e.g., Newey and Zahra, 2009), which is the “ability to self‐adapt” to rapid change (Aggarwal et al., 2017, p. 5; see also: Mahdad et al., 2020). Prior studies on exogenous shocks have approached this organizational ability from different perspectives. Among research on the role of economic resources in overcoming crises, a study on the airline industry post 9/11 found that companies with stronger economic reserves were better able to adjust to the exogenous shock (Gittell et al., 2006). Beyond economic resources, noneconomic resources also help companies cope with a crisis and adapt to it. For instance, organizations’ human resources, their cognitive abilities (Lengnick‐Hall and Beck, 2005; Lengnick‐Hall et al., 2011), and the routine of regularly sharing information inside the firm (Gittell, 2008) were found to be prerequisites for reacting flexibly to exogenous shocks.

Despite their economic relevance, there is surprisingly little scholarly attention to how family firms react to exogenous shocks. The idiosyncratic features of family firms might affect the way they react to crises. Common impediments, such as paternalistic leadership structures (Chirico et al., 2012; Soluk and Kammerlander, 2021), rigid mental models (König et al., 2013), and the reluctance to cooperate with new external partners (De Massis et al., 2015), suggest that family firms might be less inclined to adapt to crises than their nonfamily counterparts. However, extant family firm innovation research also shows that despite their resource constraints, family firms rely on noneconomic resources to remain competitive and overcome environmental change (De Massis et al., 2018a; Calabrò et al., 2019; Haynes et al., 2019; Soluk et al., 2021). In particular, family firms benefit from their efficient decision‐making processes (Duran et al., 2016), which are facilitated by their combination of ownership and management (König et al., 2013), their entrepreneurial orientation (Zahra et al., 2004), their ability to internalize and reinterpret knowledge that pertains to the family firm’s tradition (Chirico and Salvato, 2008; De Massis et al., 2016; Kotlar et al., 2020; Soluk et al., 2021), and their access to long‐serving, loyal employees (Miller and Le Breton‐Miller, 2006). This tension between lower inclination (impediments) and higher ability (enablers) in family firm innovation has been labeled the “ability and willingness paradox” (Chrisman et al., 2015a), which refers to family firms’ superior ability but lower willingness to engage in innovation.

Prior studies on family firms and exogenous shocks revealed the strong cohesion between the family, the business, and their employees and provided evidence that family firms are less likely to dismiss staff in a crisis (Block, 2010; Colombo et al., 2014; Bjuggren, 2015). Due to their noneconomic goals, family firms are even reluctant to pursue economically advantageous exit strategies in times of distress (Akhter et al., 2016; Chirico et al., 2019). Previous research labeled these noneconomic endowments as the family firm’s *socioemotional wealth* (SEW), more formally defined as the “noneconomic utility a family derives from its ownership position in a firm” (Leitterstorf and Rau, 2014, p. 751; see also Gómez‐Mejía et al., 2007), and De Massis and Rondi (2020) argue that COVID‐19 and its aftermath have put considerable strain on several SEW dimensions, leading to unique and distinctive challenges for family firms. Another study emphasized families’ “rich collective memories” as a source of family firms’ ability to cope with performance variability (Mazzelli et al., 2020, p. 2). The business family’s goal of preserving the company in the long term (Diaz‐Moriana et al., 2020) and their desire for continuity (Salvato et al., 2010; Chrisman et al., 2015b) indicate that family firms might maintain a strong need to develop adaptive capacity, particularly during crises (Stafford et al., 2013; Haynes et al., 2019). The relevance of family firms’ noneconomic goals is also emphasized by Campopiano and colleagues (2019, p. 779), who suggest that goals such as family harmony, family social status, and family and firm identity lead family firms “that prioritize these goals to absorb environmental jolts better than family firms who prioritize other goals,” thus referring to heterogeneity in family firm resilience (see also Nordqvist et al., 2014; Conz et al., 2020). Drawing on five goal categories (i.e., power and control; transgenerational value; reputation; close, enduring ties; and emotions), noneconomic goals were also found to affect the family CEO’s sensemaking in responding to discontinuous change, such as digitalization as an environmental stimulus (Kammerlander amd Ganter, 2015; see also Chrisman et al., 2015b). Although previous research reveals first insights into which family firm idiosyncrasies may affect the reaction to crises, the COVID‐19 pandemic – due to its enormous strength and interference with economic and social life – entails unique implications that we aim to explore with our study.

## 3 Method

This study’s context is the adaptation of German family firms during the shutdown induced by the COVID‐19 pandemic (De Massis et al., 2018a; Streeck et al., 2020). Since the COVID‐19 pandemic is an emerging and currently poorly understood phenomenon in innovation research, case‐based exploratory methods are most appropriate (Eisenhardt, 1989; De Massis and Kammerlander, 2020), especially when they build on a longitudinal research design that allows capturing “the temporal, unfolding nature of crises” and explains the patterns before and after an exogenous shock (Doern et al., 2018, p. 5; see also Buchanan and Denyer, 2013).

To ensure comparability we relied on the following sampling criteria: (1) family firms with substantial family influence, (2) headquartered firms in Germany, and (3) firms being active in manufacturing. The selection of the case firms was an iterative process. To reflect the heterogeneity among family firms (Kammerlander and Ganter, 2015; Campopiano et al., 2019), we considered both firms with family and nonfamily CEOs, firms with different governance structures (family involvement in the supervisory board, advisory board, and/or family council), and firms with different generations (from 1^st^ to 4^th^ generation) (see also Eisenhardt, 1989 for theoretical sampling). The study was embedded in a larger, ongoing research project, which enabled us to build on the data collected both before and after the emergence of the COVID‐19 pandemic. Table 1 summarizes the characteristics of the four case firms.

**Table 1:** Case firms and their characteristics

Case firm	Foundation	Location	Industrial sector	Employees	Revenue	Ownership structure	Generation	Management	Other family involvement
1. Alfa	1942	Germany	Rubber and Plastics	1,200	165 m Euro	100% Family Share	3rd	Family CEO	Supervisory Board, Advisory Board
2. Bravo	1947	Germany	Traction Engineering	4,000	790 m Euro	100% Family Share	4th	Non‐Family CEO	Supervisory Board, Family Council
3. Charlie	1971	Germany	Machine Construction	900	105 m Euro	100% Family Share	1st	Family CEO	Supervisory Board
4. Delta	1850	Germany	Industrial Connectivity	5,000	830 m Euro	100% Family Share	3rd	Non‐Family CEO	Supervisory Board

Note

All names of companies and interviewees are anonymized throughout the paper as confidentiality was guaranteed to all interview partners.

To collect information on the case firms, we drew on a large number of sources (Table 2), including (1) semistructured interviews with decision makers of the case firms, (2) expert interviews with specialists from other organizations, (3) observations of the case firms, and (4) further archival sources such as annual reports, press articles, corporate documents, and web archives. Before the COVID‐19 pandemic (waves 1 and 2 of data collection), we focused on questions explaining the management, decision‐making, and digital technology adoption of family firms. During the crisis (wave 3), we added questions on how the firms were reacting and adapting to the COVID‐19 pandemic.

**Table 2:** Data sources and the three waves of data collection

Case firm	1st Wave of Data Collection (March‐July 2017)			2nd Wave of Data Collection (April‐June 2018)			3rd Wave of Data Collection (February‐May 2020)		
	Internal interviews	Observations	Additional data	Internal interviews	Observations	Additional data	Internal interviews	Observations	Additional data
1. Alfa	CEO, HoX 1, Project Manager	Industry fair, corporate headquarter, trading partner	Web archives, annual reports, press releases, corporate documents	HoX 2, Manager	Industry fair, corporate headquarter	Web archives, annual report, press releases, corporate documents, brochures	Family‐CEO, CDO	Corporate headquarter	Web archives, press releases, press articles, corporate documents
2. Bravo	HoX 1, HoX 2, Manager, Specialist 1	Industry fair, corporate headquarter	Web archives, annual reports, secondary interviews, press articles, brochures, corporate documents	HoX 3, HoX 4	Industry fair, trading partner, customer	Web archives, annual report, press releases, corporate documents	Manager, Specialist 1, Specialist 2	Trading partner, corporate webinar	Web archives, press releases, press articles, blog articles, corporate documents
3. Charlie	Family‐CEO, HoX 1, HoX 2	Corporate headquarter, trading partner	Web archives, press articles, corporate documents	HoX 3	Industry fair, distributor	Web archives, annual report, brochures, press articles, corporate documents	Family‐CEO, HoX 1	Distributor	Web archives, corporate documents, press releases, press articles, brochures
4. Delta	Director, Manager 1, Manager 2	Industry fair	Web archives, press releases, brochures, chamber of commerce's report	HoX, Manager 3, Manager 4	Industry fair, corporate headquarter	Web archives, annual report, press articles, corporate documents	Director, Manager 1	Corporate headquarter	Web archives, annual report, press articles, press releases, corporate documents
Case specific data	13 Interviews	8 Observations	17 Add. Data	8 Interviews	9 Observations	18 Add. Data	9 Interviews	5 Observations	19 Add. Data
Expert interviews	23 expert interviews			16 expert interviews			21 expert interviews		

CEO, chief executive officer; CDO, chief digital officer; CTO, chief technology officer; HoX, head of department.

**Figure 1:**
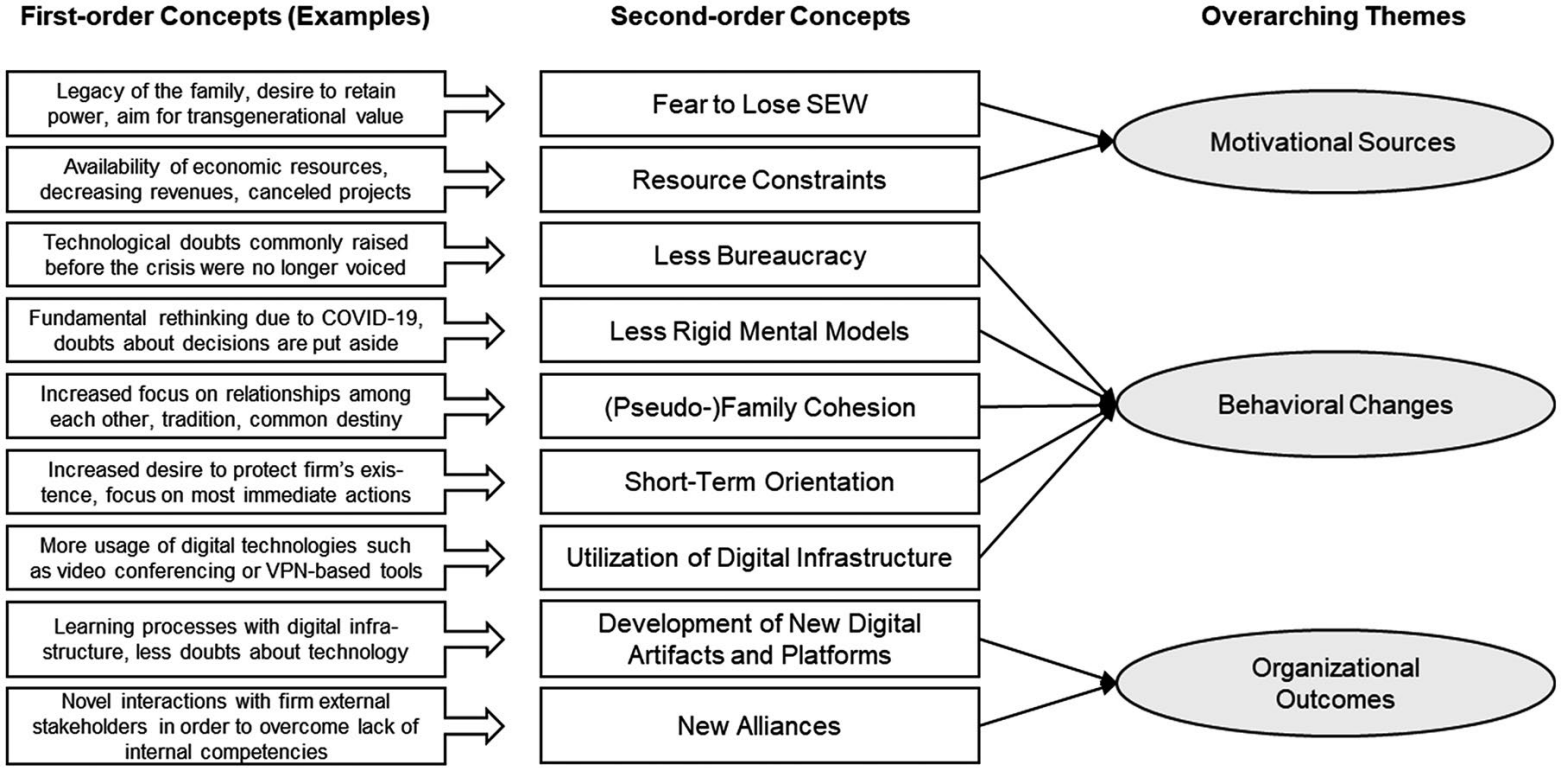
The data structure including first‐order concepts, second‐order concepts, and overarching themes.

With six to nine interviews per case firm, the inclusion of different perspectives, the use of observations and archival data, and expert interviews, we built our findings on a variety of in‐depth information sources. To analyze the data, we first created an overview of the activities before and after the shock, triangulated the different data sources, and thus mitigated potential concerns of biased responses. We coded the cases based on open coding and then moved from a within‐case to a cross‐case pattern analysis following an axial coding approach (Corbin and Strauss, 2008). Then, we stepwise developed the first‐ and second‐order concepts and overarching themes (Figure 1) as well as the framework illustrated in the subsequent sections.

## 4 Findings

**Figure 2:**
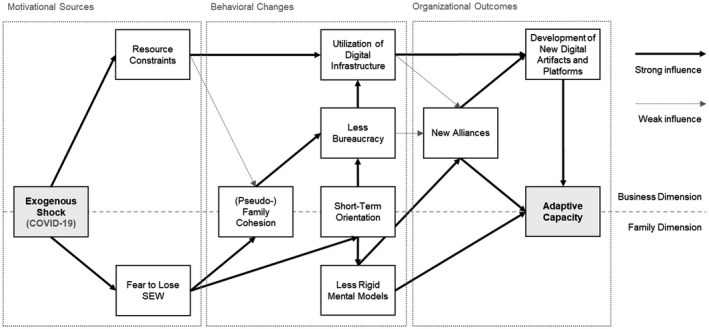
Framework of a family firm’s adaptation to an exogenous shock.

In the following, we provide an overview of the activities we observed in the case firms in association with COVID‐19. We explain motivational sources, behavioral changes, and organizational outcomes related to this exogenous shock. The study’s insights are also illustrated in Figure 2, while additional information on the case firms is offered in Table 3. In addition, Table 4 provides an overview of the heterogeneity among the case firms.

**Table 3:** Short descriptions of the four case firms

Case firm	Description
Alfa	Alfa is a German family firm – founded in 1942 – comprising 13 subsidiaries worldwide and 1,200 employees at over 20 locations (e.g., Poland, Netherlands, United States, Canada, and China). The focus of the business is to design and manufacture tailor‐made products, in particular, plastic and rubber parts for further application in various industries (e.g., mechanical engineering, environmental engineering, automotive). The management board consists of three brothers acting in the third generation as a family business. Brother A holds a doctorate in mechanical engineering and is managing director of three subsidiaries. Brother B holds a diploma in mechanical engineering and is responsible for three other subsidiaries. Brother C holds a diploma in business and management and is responsible for finance and controlling, personnel, and IT at the Alfa Holding.
Bravo	Bravo was founded in 1947 and is located with its own subsidiaries in 26 countries (e.g., all over Europe, United States, Brazil, China, Malaysia, and Russia). The company has 4,000 employees and produces power units, automation systems, frequency converters, gears and geared motors, and controls. In 1996, for the first time, a nonfamily CEO was appointed; currently, family members’ involvement is limited to their presence in the supervisory board and the family council. In 1990, the founder’s daughter established a foundation to commemorate her father’s 100th birthday, supporting young scientists for postgraduate studies, project work, and doctoral stipends. The current head of the foundation is a great‐granddaughter of the company founder.
Charlie	Charlie was founded in 1971; the founder, now 78 years old, is still managing director of the company, and today he shares the company’s ownership with three other family members. At times he was supported by (nonfamily) co‐CEOs, but since 2016 he again works as the sole family CEO. The company has 900 employees and three production sites in Germany, six subsidiaries worldwide (e.g., Great Britain, United States, Poland, and Spain) and mainly manufactures balers and document shredders in the environmental and office technology sectors. In recent years, there have been different considerations regarding handing over the company to the next generation of the family, but for various reasons, this has not yet been realized. The company has far‐above‐average in‐house production depth, wide‐ranging quality control facilities, and an export share of more than 70%.
Delta	Delta was founded in 1850 and in 1937 it was completely taken over by the ancestor of the family, who still holds all the shares of the company today in the third generation. With manufacturing locations in five countries (Germany, Czech Republic, Romania, China, and Brazil), the business with its 5,000 employees produces connection technology devices, electronic parts, and automation solutions; including sales agencies, it is represented in a total of 80 countries. Currently, the involvement of the owning family is limited to their presence in the supervisory board. Since 2013, the company has been working intensively with Industry 4.0 applications, resulting in various new offerings. The company is actively involved in several industry associations and maintains numerous research cooperations, particularly with regional partners – but also in China.

**Table 4:** Heterogeneity of selected motivational sources, behavioral changes, and organizational outcomes among the case firms

Case firm	Resource constraints	Fear to lose SEW	(Pseudo‐) family cohesion	Short‐term orientation	Less rigid mental models	New alliances	Contribution to sustained digital transformation	Adaptive capacity
Alfa	High	High (in particular legacy, power, and control, transgenerational value)	High (in particular family identity)	High	High	High	High	High
Charlie	High	High (in particular legacy, power and control, transgenerational value)	High (in particular enduring ties, power, and control)	High	Medium	High	Medium/High	High
Bravo	Medium/High	High (in particular legacy, transgenerational value)	Medium/High (in particular family identity)	Medium/High	Medium	Medium	Medium	Medium/High
Delta	Medium/High	High (in particular transgenerational value)	Medium/High (in particular family identity)	Medium/High	Medium	Low	Medium	Medium

### 4.1 The exogenous shock and motivational sources

Before the COVID‐19 shock, the case firms displayed heterogeneous degrees of family involvement, governance structures, and generations in control, inducing different nuances in their behavioral patterns. While they were all characterized by the influence of the owning family, such influence was particularly strong – with a thorough involvement in daily business routines – for the case firms with a family CEO (Alfa, Charlie). While Bravo (family firm with a nonfamily CEO) showed moderate family involvement through its family council, which was set up to support strategic issues, Delta (family firm with a nonfamily CEO) showed the weakest involvement of the family in the business, with family members acting in a rather overseeing role on the supervisory board. In its various forms, family involvement has consistently led to a long‐term orientation and a desire for continuity in firms. These attributes were particularly strong in Alfa and Charlie. Charlie’s head of department emphasized this strategic focus as follows:

“The planning horizons are always in the long run, [the CEO and owner] attached great importance to the fact that we developed continuously and positively. Quarterly figures did not play a major role if the long‐range picture was accurate.”

Alfa’s family CEO underlined their long‐term focus and desire for continuity:

“We want to preserve the company as a family and create sustainable value across generations.”

However, these organizational patterns were also omnipresent in the two other family firms, as is reflected in the interviews and secondary data. Bravo mentions the term “long‐term” 24 times in its annual report 2018/2019, Delta mentions “long‐term investments” (besides “building up internal know‐how”) in a press release on the annual report 2019 as the most important key factor for future success.

Although the case firms regularly engaged in new product development before the COVID‐19 outbreak, and thus built up a solid market position, some signs of organizational inertia became evident over time. This was particularly, but not exclusively, evident for the two firms with a family CEO (Alfa, Charlie). For instance, interviewees at Charlie reported considerable paternalistic patterns based on the founder’s leadership style that impeded the development of (digital) innovation projects. The 78‐year‐old family CEO – together with long‐standing partners and aiming to strengthen family reputation – has placed successful products on the market during his almost 50 years of activity. However, doubts were growing, especially in the past ten years, as digital innovations that could have helped to keep up with the increasingly globalized world market remained rare. As a result, the company’s competitive position has deteriorated. Charlie’s head of department explained that the family CEO rejects digital innovation projects because he is not sensitive to digital technologies and never truly used them himself. We identified similar barriers for Alfa. The project manager responsible for digitalization explained that tensions between the three brothers involved in the management are a major challenge:

“As it is in a family business, brothers can hardly agree [on digital projects]. [...] [When it comes to enacting and implementing digital projects], there is usually no agreement on the issues.”

In addition to paternalistic leadership structures and rigid mental models that hampered decision‐making, the case evidence of Alfa and Charlie revealed serious obstacles to working with new external business partners (by adhering to established long‐standing relationships), impeding innovation in those firms. For Bravo and Delta, we also observed similar organizational patterns but to a lower extent due to their different governance structures and limited family involvement.

All four case firms had in common that the COVID‐19 pandemic triggered a radical environmental change. While three case firms (Alfa, Bravo, Charlie) were able to anticipate the crisis at an early stage, Delta was fully hit once the lockdown was enacted in Germany. Alfa’s family CEO emphasized the following:

“We have a branch in Shenzhen, China, for which we had to develop a local protection plan in January. Because we are so internationally oriented, we anticipated quite early what was going on.”

What was common for all companies, however, was the feeling that a fundamental change had occurred within a short period of time, that is, the way in which the business was previously conducted was no longer possible after this exogenous shock. Charlie’s family CEO underlined:

“The whole company is affected by the coronavirus, and all of a sudden, almost all the markets have collapsed without us being able to do anything about it.”

Firm representatives reported a “lethargic” condition that temporarily prevailed in their organizations (regardless of family involvement). This condition was expressed by suspending business decisions to first gather new information on the crisis. The lack of experience with such a pandemic led to disorientation for a short period of time.

Regarding shifts triggered by the exogenous shock, we identified two patterns in our cross‐case analysis; one pattern relates to the availability of economic resources in the company. Governmental lockdowns caused a massive drop in revenues during the crisis because the existing value chains could not be maintained. Delta’s director explained it as follows:

“It quickly became clear what a huge impact COVID‐19 has on our industry and our customers – and therefore on us [following a demand shock]. We had budget cuts and the freezing of cost‐intensive projects to secure liquidity. As of this May, we are now also working short‐term at our company.”

In the business dimension, the sudden loss of most revenues for all four case firms led to acerbated resource constraints. Among others, this economic outcome was reflected in the discontinuation of corporate projects and the shortening of budgets. Due to their tendency to rely on external financiers only in exceptional situations and otherwise finance themselves through profit retention (which made large‐scale investments such as those observed in public companies very difficult), the studied family firms were already resource constrained *before* the external shock, which was exacerbated during the crisis. In Bravo and Delta, which were more used to working with debt capital than the other two firms with family CEOs, the interviewees reported that access to government support loans and grants was somewhat easier. This led to a more rapid alleviation of the strained liquidity situation, although the government loans and grants were not able to compensate for the collapsing revenues. In the end, due to their lack of experience with external financiers, the economic constraints on Alfa and Charlie were particularly severe during the COVID‐19 pandemic.

As the second pattern, referring to the family dimension, the exogenous shock had an impact on a diverse set of noneconomic motivations. With regard to the family dimension, we observed that the owning family was exposed to a fear of SEW loss due to the crisis, which was mainly related to the legacy of the family business (particularly evident in Alfa, Bravo, and Charlie as the firms with governance structures allowing the strongest family involvement), the desire to retain power and control (particularly evident in Alfa and Charlie with their family CEOs), and the aim to create transgenerational value (evident in all four firms). As Charlie’s head of department revealed:

“We noticed that the family management was very concerned about the continuation of the business.”

Alfa’s family CEO emphasized the following:

“Of course, this has kept us increasingly busy, not only in a business sense but also emotionally.”

The owning family was afraid of losing their socioemotional endowments in the crisis. This fear was reinforced by the prolonged uncertainty regarding the course of the COVID‐19 pandemic. Based on our analysis, we thus propose the following:

Proposition 1 *An exogenous shock triggers a) economic implications in the business dimension (*i.e.*, reinforced resource constraints) and b) noneconomic implications in the family dimension (*i.e*., increased fear of losing SEW) in the family firm. While the noneconomic implications in the family dimension are high in all case firms, the business implications are particularly high in more resource‐constrained firms*.

### 4.2 Behavioral changes in times of crisis

Our cross‐case pattern analysis revealed how the motivational changes in increased resource constraints and increased fear of losing SEW induced behavioral changes in firm decision makers. Case evidence indicates that SEW‐loss‐related anxieties affect not only the family but also the workforce. The strong relationships among each other, the shared tradition as a family business, and the feeling of having a “common destiny” led to a particular strong cohesion within the family but also among the workforce, which feels like a “pseudofamily.” Interestingly, this effect became evident in all four firms but was explained differently by the interviewees. For Alfa (3^rd^ generation), Bravo (4^th^ generation), and Delta (3^rd^ generation), the long‐standing family identity – existing over several generations – was the essential criterion (even for nonfamily employees whose parents and grandparents have often worked in the same company) for developing a (pseudo‐)family cohesion. For Charlie, it was rather the tight connection of the founder and family CEO with the firm’s daily business routines. Hence, strong (pseudo)family cohesion was driven by noneconomic goals such as the aim to maintain ties to stakeholders, perpetuate the emotional endowment between the (pseudo)family and the business, and a strong desire to maintain power and control, ultimately inducing an increased focus on *all* organizational members and cohesion within the firms. From the beginning of the COVID‐19 pandemic (i.e., March 2020), the (family and nonfamily) managers of the case firms communicated that the well‐being of the employees was their primary concern. For instance, an internal letter from Bravo states that the protection of employees “is a top priority and is at the forefront of the extensive action plan that we have consistently implemented throughout the company since the beginning of the pandemic,” emphasizing the strong ties between family, management, and employees. Although many employees have also suffered personal economic losses as a result of short‐term working, this German state‐regulated system has helped firms avoid laying off any firm employees. As Bravo’s manager commented:

The family has behaved calmly and has treated the staff well. The crisis brought us closer together. This cohesion has also led to the fact that we have come through it reasonably well so far.

Further, we observed that the severe resource constraints – because of the shared concern for the company’s survival – also brought the (pseudo‐)family closer together. As a consequence of this (pseudo)family cohesion, we noted in all case firms a flexible, sudden “hands‐on mentality” in regard to responding to the critical situation. In the business dimension, this mentality led to a reduction in bureaucratic hurdles and quicker decisions when such decisions had the aim of mitigating the effects of the crisis. Alfa’s family CEO stated the following:

I think it’s good that we, as a family business in the third generation, have a good view of the business and know what to do. We know the people in charge and can thus intervene quickly without long hesitation and, if necessary, take action.

In addition, the worries related to SEW loss shifted the strategic focus of the firms. The owning families have moved away from the former long‐term orientation that we observed before the COVID‐19 pandemic. In contrast to previous times, the families started to particularly focus on the short‐term results of their actions. In Alfa and Charlie, this effect was perceived quickly and distinctly due to their family CEOs, but in Bravo and Delta, this development became noticeable. The reason for this approach was to protect the existence of the company in times of extreme uncertainty. Charlie’s head of department explained as follows:

We quickly put our focus on tackling all the hot issues that needed to be dealt with on short notice and with which we could ensure the safety of the workforce.

In the family dimension, the focus on short‐term results mitigated the rigid mental models that business families – particularly, although not exclusively, Alfa and Charlie with their family CEOs – exhibited before the crisis. In particular, these family CEOs, as well as other family members, underwent fundamental rethinking due to COVID‐19. Doubts about decisions were put aside if the decisions appeared to help the company immediately. Delta’s director described these changes as follows: “[The owning family] is seen as support to overcome this crisis. Attitudes have changed for the better.” Even Charlie’s family CEO, who had previously shown no affinity for digitalization and remote working and who had demonstrated extensive rigid mental models before the exogenous shock, cleared the way at short notice and revealed new ways of thinking. He stated:

Wherever possible, the administrative staff is equipped to work from home – we have made this possible at short notice, that is not a question at all.

Building on that, in the business dimension, the case evidence revealed behavioral changes relating to the use of technologies in everyday routine. As economic resources became increasingly scarce during the crisis, operational procedures were critically scrutinized. Case firms reported that business trips, educational trainings, trade fair exhibitions, and projects were canceled. As a resource‐saving alternative, a switch to a digital infrastructure with video conferencing tools or virtual private network (VPN) servers to enable home offices was implemented in many areas. This extended the utilization of digital infrastructures was initially necessity‐driven, arising from the crisis situation and contact restrictions. In a press article, Delta’s CEO stated that in April 2020, 2,000 out of 2,300 employees in Germany were working from home. A manager from Delta explained in more depth:

We switched to digital working, at least where possible. [Because of the pandemic], we have basically digitized almost every form of normal interaction.

Furthermore, we observed that the lower bureaucratic requirements within the company contributed to building a new digital infrastructure. The technological doubts that were common before the exogenous shock – particularly, but not exclusively, in Alfa and Charlie – were no longer voiced during the crisis. Based on those observations, we present the following proposition:

Proposition 2 *The fear of losing SEW triggers (pseudo‐)family cohesion and short‐term orientation, leading to less rigid mental models in the business family and less bureaucracy in the family business. These implications, in combination with increasing resource constraints, foster an expansion of the digital business infrastructure. The respective characteristics are particularly evident in case firms with a family CEO and strong family influence*.

### 4.3 Organizational outcomes and adaptive capacity

Building on the behavioral changes induced by COVID‐19, we observed specific organizational outcomes in our case firms. In the business dimension and due to the state of distress, these behavioral changes led the family firms to open themselves up to new alliance partners. While before the exogenous shock, the family firms found it difficult to cooperate with new external partners and were more focused on internal developments (particularly Alfa and Charlie with their family CEOs’ desire to maintain power and control as well as their aim to sustain their long‐established and enduring ties), we observed an opposite trend during the pandemic. The family firms increasingly started to cooperate with local networks, governmental agencies, new suppliers, and partially with start‐ups. During the pandemic, family firm decision makers realized that not all competencies required to overcome the exogeneous shock were available internally. A director at Delta stated:

We use new software and suppliers to [...] increase flexibility. [...] However, we are also working with new – often highly specialized – cooperation partners in sales and health protection.

In the expert interviews, this trend was confirmed by suppliers who cooperated with the case firms and other companies. A senior manager from an IT service provider explained in an expert interview:

The demand for IT solutions has increased rapidly due to the pandemic. [...] Family businesses were much more reluctant [regarding IT] before the crisis, their purchasing habits and collaboration attitudes changed drastically due to COVID‐19.

The aforementioned behavioral changes have also yielded novel approaches to innovation in the business dimension. We observed that the four firms were in a substantial learning process at the time they utilized their expanded digital infrastructure. After the first weeks of the pandemic, certain routines with digital work procedures had already been developed, and thus, previous doubts regarding technology adoption were dispelled. This increased experience in handling digital technologies also enabled the firms to develop digital artifacts, such as digital services, products, business models, and platforms, to be offered to customers for two reasons. First, because they were now technologically capable of doing so, and second, because the contact restrictions prevented many conventional services and business models from being provided. Despite the continuing limitations, the development of these new digital artifacts and platforms was not only necessity‐driven (as in the case of the digital infrastructure), but its motivation was also opportunity‐driven. As Bravo’s manager explained:

We have just started a webcast [...]. There, we provide a regular overview of certain sales topics [...], where we virtually meet with customers and other interested parties. Not only do we present current trends, we also engage in an interactive exchange with the participants and discuss where possible fields of application are. For me, this is a good example of how to turn necessity into virtue and find a good answer to canceled trade fairs by digital means.

Case evidence revealed that Bravo and Delta, with their nonfamily CEOs, one of whom had already managed digitalization projects in other companies and brought professional experience with him, were able to develop new digital artifacts and platforms relatively fast. We also observed that the new alliance partners facilitated the development of new digital artifacts and digital platforms. This access to external knowledge enabled the firms to complement the internal competencies required for digital artifacts and platforms.

Observations during the COVID‐19 pandemic also revealed three particular factors that ultimately helped family firms adapt to the exogenous shock. We label this improved position to self‐adapt to the crisis the “*adaptive capacity*” the firms developed after the COVID‐19 outbreak. First, digital artifacts and digital platforms allowed the case firms to become flexible and to react quickly to changing market requirements, thus resulting in increased adaptive capacity. Second, the development of new strategic alliances had a considerable impact on the adaptive capacity of the family firms that can now choose from a broader portfolio of cooperation partners, develop complementary market approaches, and act flexibly in a business ecosystem. Regarding the relationship between digital artifacts, alliances, and a family firm’s adaptive capacity, Charlie’s head of department mentioned the following:

I hope we can use some of the points [...] for future benefit. For instance, [...] the facilitated digitalization and robust business relationships. If we manage to sustain the progress that has been achieved despite the chaos triggered by this crisis, then we will have gained something.

Third, it is the mental rethinking of the business family that has facilitated the firms’ adaptive capacity, hence affecting both the business and the family dimension. Using the family’s influence for continuous adaptation in crisis situations and beyond, therefore, turns out to be beneficial for the business. As Delta’s director stated,

Stubbornness and insisting on old structures is deadly in the corona crisis, and no one can afford it. Instead, I regard flexibility in dealing with changes, on a small scale, as well as in the case of major disasters, such as the corona crisis, as an essential success factor.

Based on the cross‐case pattern analysis, all these influences, in combination, reveal that family firms can cope with exogenous shocks to build up adaptive capacity and succeed after the crisis. Revealing the heterogeneity of the four case firms, we see that Alfa and Charlie, with their lower degrees of rigid mental models, higher degrees of new alliances, and development of new digital artifacts and platforms, ultimately yield higher levels of adaptive capacity than Bravo and Delta, where these antecedent conditions were slightly less salient. This leads us to propose the following:

Proposition 3 *The development of new digital artifacts, digital platforms, and new strategic alliances, in combination with less rigid mental models, facilitates the adaptive capacity of family firms. Different degrees of these aforementioned antecedent conditions ultimately yield heterogeneous levels of family firms’ adaptive capacity*.

## 5 Discussion

Our findings reveal that exogenous shocks induce behavioral changes and thus generate organizational outcomes, which ultimately facilitate a family firm’s adaptive capacity. Our research not only advances extant theory, as discussed below, but also provides important insights for management practice and policymaking.

Our findings first contribute to research on exogenous shocks by revealing how organizations react to crises and adapt to them. By revealing how drastic and survival‐threatening the impact of exogenous shocks is for businesses, we find empirical support for phenomena (such as financial crises or major terrorist attacks) described by prior studies in this field (Smallbone et al., 2012; Corbo et al., 2016; Doern et al., 2018). However, ours is one of the first studies to explore the effect of the COVID‐19 pandemic on business management, thereby advancing the current understanding of this phenomenon and its reverberations. By explaining the underlying patterns of organizations’ adaptation to exogenous shocks, we extend the prevailing insights on crises and adaptations, as prior studies often lack an understanding of microissues (Chakrabarti, 2015). In particular, we contribute to a nuanced understanding of how heterogeneous noneconomic goals can affect the response of firms to an exogenous shock. We also extend research on adaptive capacity and environmental change (Aggarwal et al., 2017). We do this particularly by revealing the specific behavioral changes and organizational outcomes induced by an exogenous shock, which ultimately lead to a firm’s adaptive capacity.

Another important contribution of our study addresses the family business literature, specifically related to innovation management and adaptive capacity in family firms. Our findings support the existence of resource constraints (Feranita et al., 2017; De Massis et al., 2018a), the importance of SEW and noneconomic goals in family firms (Gómez‐Mejía et al., 2007; Berrone et al., 2012), and the vital role of a family firm’s tradition in innovation processes (De Massis et al., 2016; Erdogan et al., 2020). However, by showing how these family firm idiosyncrasies are reinforced in times of crisis and induce heterogeneous responses, we extend this previous understanding. We unveil that due to an exogenous shock and family firms’ tendency for self‐financing, resource constraints are particularly obstructive in firms with a family CEO (goal: retain power and control). This advances research that identified heterogeneity as an important direction for future scholarship, while remaining silent about explanations of how diverse family firms’ characteristics may affect resource constraints (De Massis et al., 2018a). By showing that family firms with governance structures allowing strong family involvement (e.g., family management, family council, advisory board) are particularly concerned about losing SEW (goals: preserve the legacy of the family, retain power and control, and create transgenerational value), we enrich previous SEW research that has so far largely neglected to account for different types of family involvement and link them to different propensities for SEW preservation (Gómez‐Mejía et al., 2007; Berrone et al., 2012; Miller and Le Breton‐Miller, 2014). With these insights, we extend prior SEW research by showing that varying types of family involvement exerted through different governance structures affect SEW in heterogeneous ways (Li and Daspit, 2016). Moreover, by revealing that (pseudo‐)family cohesion is particularly strong for family firms in a later (3^rd^ or 4^th^) generation or with a hands‐on founder figure (goals: enduring ties to stakeholders, emotions and affect, and family reputation), we extend previous findings that highlighted (pseudo‐)family cohesion in family firms but remained silent about its heterogeneity among family firms (König et al., 2013; Duran et al., 2016). With the aforementioned insights, we contribute to family business research on heterogeneity and reveal insights into specific noneconomic goals in those firms (Kammerlander and Ganter, 2015; Campopiano et al., 2019). Research on family firms has stressed that these businesses are usually characterized by long‐term orientation (Diaz‐Moriana et al., 2020), rigid mental models that are often associated with paternalistic leadership styles (König et al., 2013), higher ability yet lower willingness to engage in technological innovation (Chrisman et al., 2015a), which is linked to an inward focus on technological developments (De Massis et al., 2015) and difficulties in utilizing digital technologies (Soluk and Kammerlander, 2021). Challenging this body of knowledge, we reveal that these aspects are dependent on environmental conditions (Soluk et al., 2021) and can be dramatically altered by exogenous shocks. In times of crisis, family firms may exhibit a short‐term orientation, reject rigid mental models and become more willing to engage in technological innovation, create new forms of cooperation, and unbureaucratically develop new digital business opportunities. With these insights, in addition to revealing how crises are related to intensifying search routines (Mazzelli et al., 2020), creating new alliances, and open innovation approaches (Chiesa and Manzini, 1998; Classen et al., 2012; Casprini et al., 2017), we also offer a more nuanced understanding of how family firms develop adaptive capacity. In addition, by studying family firms that *anticipate* a future loss (rather than experiencing one), we advance the understanding of decision making and innovation under different domains (i.e., loss and gain domains; Chrisman and Patel, 2012). Specifically, based on our study’s findings, family firm status seems to foster a particularly proactive reaction to a threat.

Our study also provides practical implications. With our in‐depth insights, we provide nuanced explanations of how, in particular, family firms adapt to exogenous shocks. By revealing the specific motivational sources, behavioral changes, and organizational outcomes associated with a family firm’s adaptation to an exogenous shock, we provide practitioners with knowledge on how they can foster the adaptive capacity of their company and thus ensure long‐term success beyond crises. More specifically, based on our study’s insights, practitioners can build on the revealed enablers of a firm’s adaptive capacity (e.g., new alliances or digital technologies) and ensure that the actual conditions in the organizations allow these enablers to emerge (e.g., with the elimination of regulatory hurdles). Additionally, we contribute to a better understanding of exogenous shocks and their effect on policymaking (De Massis et al., 2020). Due to the crucial role of government support both during and after crises (Burrell and Kelly, 2020), the insights from our study inform policymakers about how to enact more targeted interventions to mitigate the negative economic impact of such crises (e.g., by creating institutional conditions that allow new alliances to emerge). In light of their key role in any economy worldwide, we believe that considering the idiosyncrasies of family firms is particularly critical for the success of policymaking initiatives aimed at supporting businesses and economic development (Brautzsch et al., 2015).

## 6 Conclusion, limitations, and future research

With an in‐depth explanation of how exogenous shocks affect family firms, we reveal which patterns occur in those businesses when hit by the COVID‐19 pandemic. This study contributes to research, management practice, and policymaking by advancing our knowledge on the idiosyncrasies of family firms. As with all qualitative studies, our research entails limitations, which offer promising opportunities for future research. First, with our multicase study, we can only claim analytic generalization; however, we hope to stimulate future research to scrutinize our case‐based findings with large‐scale, quantitative approaches and investigate whether and how the findings might be generalized statistically (e.g., to other firm types or contexts). As the effects of the COVID‐19 pandemic persisted at the time this study was finalized, we would like to encourage scholars to examine organizations’ behavior in the aftermath of such a crisis, thus extending the limited time window of observation. Additionally, although we followed established guidelines on the number of cases for our study (Eisenhardt, 1989), we encourage future scholars to build on a broader set of cases to delve deeper into explaining the heterogeneity of family firms based on attributes other than those on which we focused (family management, governance structure, generation). Moreover, additional theoretical approaches, such as prospect theory (Tversky and Kahneman, 1974), might act as suitable lenses to explain behavioral changes in the loss domain, as revealed in our study. We hope that the findings presented in this article will stimulate future work to further examine how organizations succeed through exogenous shocks and better understand the determinants of their resilience.

## References

[radm12471-bib-0001] Aggarwal, V.A. , Posen, H.E. , and Workiewicz, M. (2017) Adaptive capacity to technological change: a microfoundational approach. Strategic Management Journal, 38(6), 1212–1231.

[radm12471-bib-0002] Akhter, N. , Sieger, P. , and Chirico, F. (2016) If we can’t have it, then no one should: shutting down versus selling in family business portfolios. Strategic Entrepreneurship Journal, 10(4), 371–394.

[radm12471-bib-0003] Berrone, P. , Cruz, C. , and Gómez‐Mejía, L.R. (2012) Socioemotional wealth in family firms: theoretical dimensions, assessment approaches, and agenda for future research. Family Business Review, 25(3), 258–279.

[radm12471-bib-0004] Bjuggren, C.M. (2015) Sensitivity to shocks and implicit employment protection in family firms. Journal of Economic Behavior & Organization, 119, 18–31.

[radm12471-bib-0005] Block, J. (2010) Family management, family ownership, and downsizing: evidence from S&P 500 firms. Family Business Review, 23(2), 109–130.

[radm12471-bib-0006] Bornheim, S. (2000) The Organizational Form of Family Business. Boston, MA: Kluwer Academic Publishers.

[radm12471-bib-0007] Brautzsch, H.‐U. , Günther, J. , Loose, B. , Ludwig, U. , and Nulsch, N. (2015) Can R&D subsidies counteract the economic crisis? – macroeconomic effects in Germany. Research Policy, 44(3), 623–633.

[radm12471-bib-0008] Buchanan, D.A. and Denyer, D. (2013) Research tomorrow’s crisis: methodological innovations and wider implications. International Journal of Management Reviews, 15(2), 205–224.

[radm12471-bib-0009] Burrell, R. and Kelly, C. (2020) The COVID‐19 pandemic and the challenge for innovation policy. Northern Ireland Legal Quarterly, 71(1), 89–94.

[radm12471-bib-0010] Calabrò, A. , Vecchiarini, M. , Gast, J. , Campopiano, G. , De Massis, A. , and Kraus, S. (2019) Innovation in family firms: a systematic literature review and guidance for future research. International Journal of Management Reviews, 21(3), 317–355.

[radm12471-bib-0011] Campopiano, G. , De Massis, A. , and Kotlar, J. (2019) Environmental jolts, family‐centered non‐economic goals, and innovation: a framework of family firm resilience. In: Memili, E. and Dibrell, C. (Eds), The Palgrave Handbook of Heterogeneity among Family Firms. Cham: Palgrave Macmillan, pp. 773–789.

[radm12471-bib-0012] Casprini, E. , De Massis, A. , Di Minin, A. , Frattini, F. , and Piccaluga, A. (2017) How family firms execute open innovation strategies: the Loccioni case. Journal of Knowledge Management, 21(6), 1459–1485.

[radm12471-bib-0013] Chakrabarti, A. (2015) Organizational adaptation in an economic shock: the role of growth reconfiguration. Strategic Management Journal, 36(11), 1717–1738.

[radm12471-bib-0014] Chiesa, V. and Manzini, R. (1998) Organizing for technological collaborations: a managerial perspective. R&D Management, 28(3), 199–212.

[radm12471-bib-0015] Chirico, F. , Gómez‐Mejia, L.R. , Hellerstedt, K. , Withers, M. , and Nordqvist, M. (2019) To merge, sell, or liquidate? Socioemotional wealth, family control, and the choice of business exit. Journal of Management, 46(8), 1342–1379. 10.1177/0149206318818723

[radm12471-bib-0016] Chirico, F. , Nordqvist, M. , Colombo, G. , and Mollona, E. (2012) Simulating dynamic capabilities and value creation in family firms: Is paternalism an “asset” or a “liability”? Family Business Review, 25(3), 318–338.

[radm12471-bib-0017] Chirico, F. and Salvato, C. (2008) Knowledge integration and dynamic organizational adaptation in family firms. Family Business Review, 21(2), 169–181.

[radm12471-bib-0018] Chrisman, J.J. , Chua, J.H. , De Massis, A. , Frattini, F. , and Wright, M. (2015a) The ability and willingness paradox in family firm innovation. Journal of Product Innovation Management, 32(3), 310–318.

[radm12471-bib-0019] Chrisman, J.J. , Fang, H. , Kotlar, J. , and De Massis, A. (2015b) A note on family influence and the adoption of discontinuous technologies in family firms. Journal of Product Innovation Management, 32(3), 384–388.

[radm12471-bib-0020] Chrisman, J.J. and Patel, P.C. (2012) Variations in R&D investments of family and nonfamily firms: behavioral agency and myopic loss aversion perspectives. Academy of Management Journal, 55(4), 976–997.

[radm12471-bib-0021] Classen, N. , Van Gils, A. , Bammens, Y. , and Carree, M. (2012) Accessing resources from innovation partners: the search breadth of family SMEs. Journal of Small Business Management, 50(2), 191–215.

[radm12471-bib-0022] Colombo, M.G. , De Massis, A. , Piva, E. , Rossi‐Lamastra, C. , and Wright, M. (2014) Sales and employment changes in entrepreneurial ventures with family ownership: empirical evidence from high‐tech industries. Journal of Small Business Management, 52(2), 226–245.

[radm12471-bib-0023] Conz, E. , Lamb, P.W. , and De Massis, A. (2020) Practicing resilience in family firms: an investigation through phenomenography. Journal of Family Business Strategy, 11(2), 1–16.

[radm12471-bib-0024] Corbin, J. and Strauss, A. (2008) Basics of Qualitative Research: Techniques and Procedures for Developing Grounded Theory. Thousand Oaks, CA: Sage Publications Ltd.

[radm12471-bib-0025] Corbo, L. , Corrado, R. , and Ferriani, S. (2016) A new order of things: network mechanisms of field evolution in the aftermath of an exogenous shock. Organization Studies, 37(3), 323–348.

[radm12471-bib-0026] Craven, M. , Liu, L. , Mysore, M. , and Wilson, M. (2020) COVID‐19: implications for business. Accessed 18 April 2020 from https://www.aedcr.com/sites/default/files/docs/mckinsey‐full_article.pdf.pdf.pdf

[radm12471-bib-0027] Davidsson, P. and Gordon, S.R. (2016) Much ado about nothing? The surprising persistence of nascent entrepreneurs through macroeconomic crisis. Entrepreneurship Theory and Practice, 40(4), 915–941.

[radm12471-bib-0028] De Massis, A. , Audretsch, D. , Uhlaner, L. , and Kammerlander, N. (2018a) Innovation with limited resources: management lessons from the German Mittelstand. Journal of Product Innovation Management, 35(1), 125–146.

[radm12471-bib-0029] De Massis, A. , Di Minin, A. , and Frattini, F. (2015) Family‐driven innovation: resolving the paradox in family firms. California Management Review, 58(1), 5–19.

[radm12471-bib-0030] De Massis, A. , Frattini, F. , Messeni Petruzzelli, A. , Kotlar, J. , and Wright, M. (2016) Innovation through tradition: Lessons from innovative family businesses and directions for future research. Academy of Management Perspectives, 30(1), 93–116.

[radm12471-bib-0031] De Massis, A. and Kammerlander, N. (2020) Handbook of Qualitative Research Methods for Family Business. Cheltenham Glos, UK: Edward Elgar. ISBN 978‐1‐78811‐644‐2.

[radm12471-bib-0032] De Massis, A. and Rondi, E. (2020) Covid‐19 and the future of family business research. Journal of Management Studies, 57(8), 1727–1731.

[radm12471-bib-0033] De Massis, A. , Di Minin, A. , Marullo, C. , Rovelli, P. , Tensen, R. , Carbone, A. , and Crupi, A. (2020) How the “EU Innovation Champions” successfully absorbed and reacted to the shock caused by the COVID‐19 pandemic, JRC Working Papers on Corporate R&D and Innovation No 06/2020, *European Commission*, Seville, JRC121856.

[radm12471-bib-0034] Diaz‐Moriana, V. , Clinton, E. , Kammerlander, N. , Lumpkin, G.T. , and Craig, J.B. (2020) Innovation motives in family firms: a transgenerational view. Entrepreneurship Theory and Practice, 44(2), 256–287.

[radm12471-bib-0035] Doern, R. , Williams, N. , and Vorley, T. (2018) Special issue on entrepreneurship and crises: business as usual? An introduction and review of the literature. Entrepreneurship & Regional Development, 31(5–6), 400–412.

[radm12471-bib-0036] Duran, P. , Kammerlander, N. , van Essen, M. , and Zellweger, T. (2016) Doing more with less: innovation input and output in family firms. Academy of Management Journal, 59(4), 1224–1264.

[radm12471-bib-0037] Dutton, J.E. (1986) The processing of crisis and non‐crisis strategic issues. Journal of Management Studies, 23(5), 501–517.

[radm12471-bib-0038] Eisenhardt, K.M. (1989) Building theories from case study research. Academy of Management Review, 14(4), 532–550.

[radm12471-bib-0039] Erdogan, I. , Rondi, E. , and De Massis, A. (2020) Managing the tradition and innovation paradox in family firms: a family imprinting perspective. Entrepreneurship Theory and Practice, 44(1), 20–54.

[radm12471-bib-0040] Feranita, F. , Kotlar, J. , and De Massis, A. (2017) Collaborative innovation in family firms: past research, current debates and agenda for future research. Journal of Family Business Strategy, 8(3), 137–156.

[radm12471-bib-0041] Fligstein, N. and McAdam, D. (2011) Toward a general theory of strategic action fields. Sociological Theory, 29(1), 1–26.

[radm12471-bib-0042] Gilbert, D.R. Jr . (1992) Strategy through process and the problem of strategic management. The Ruffin Series in Business Ethics, 82–96.

[radm12471-bib-0043] Gittell, J.H. (2008) Relationships and resilience care provider responses to pressures from managed care. Journal of Applied Behavioral Science, 44(1), 25–47.

[radm12471-bib-0044] Gittell, J.H. , Cameron, K.S. , Lim, S. , and Rivas, V. (2006) Relationships, layoffs, and organizational resilience. Journal of Applied Behavioral Science, 42(3), 300–329.

[radm12471-bib-0045] Gómez‐Mejía, L.R. , Haynes, K.T. , Núñez‐Nickel, M. , Jacobson, K.J.L. , and Moyano‐Fuentes, J. (2007) Socioemotional wealth and business risks in family controlled firms: evidence from Spanish olive oil mills. Administrative Science Quarterly, 52(1), 106–137.

[radm12471-bib-0046] Haynes, G.W. , Danes, S.M. , Schrank, H.L. , and Lee, Y. (2019) Survival and success of family‐owned small businesses after hurricane Katrina: impact of disaster assistance and adaptive capacity. Journal of Contingencies and Crisis Management, 27(2), 130–144.

[radm12471-bib-0047] Hermann, C.F. (1963) Some consequences of crisis which limit the viability of organizations. Administrative Science Quarterly, 8(1), 61–82.

[radm12471-bib-0048] Ivanov, D. (2020) Predicting the impacts of epidemic outbreaks on global supply chains: a simulation‐based analysis on the coronavirus outbreak (COVID‐19/SARS‐CoV‐2) case. Transportation Research Part E: Logistics and Transportation Review, 136, 101922. 32288597 10.1016/j.tre.2020.101922 PMC7147532

[radm12471-bib-0049] Kammerlander, N. and Ganter, M. (2015) An attention‐based view of family firm adaptation to discontinuous technological change: exploring the role of family CEOs' noneconomic goals. Journal of Product Innovation Management, 32(3), 361–383.

[radm12471-bib-0050] König, A. , Kammerlander, N. , and Enders, A. (2013) The family innovator's dilemma: how family influence affects the adoption of discontinuous technologies by incumbent firms. Academy of Management Review, 38(3), 418–441.

[radm12471-bib-0051] Kotlar, J. , De Massis, A. , Frattini, F. , and Kammerlander, N. (2020) Motivation gaps and implementation traps: the paradoxical and time‐varying effects of family ownership on firm absorptive capacity. Journal of Product Innovation Management, 37(1), 2–25.

[radm12471-bib-0052] Kuckertz, A. , Brändle, L. , Gaudig, A. , Hinderer, S. , Reyes, C.A.M. , Prochotta, A. , Steinbrink, K.M. , and Berger, E.S.C. (2020) Startups in times of crisis – a rapid response to the COVID‐19 pandemic. Journal of Business Venturing Insights, 13, e00169.

[radm12471-bib-0053] Leitterstorf, M.P. and Rau, S.B. (2014) Socioemotional wealth and IPO underpricing of family firms. Strategic Management Journal, 35(5), 751–760.

[radm12471-bib-0054] Lengnick‐Hall, C.A. and Beck, T.E. (2005) Adaptive fit versus robust transformation: how organizations respond to environmental change. Journal of Management, 31(5), 738–757.

[radm12471-bib-0055] Lengnick‐Hall, C.A. , Beck, T.E. , and Lengnick‐Hall, M.L. (2011) Developing a capacity for organizational resilience through strategic human resource management. Human Resource Management Review, 21(3), 243–255.

[radm12471-bib-0056] Li, Q. , Guan, X. , Wu, P. , Wang, X. , Zhou, L. , Tong, Y. , Ren, R. , Leung, K.S.M. , Lau, E.H.Y. , Wong, J.Y. , Xing, X. , Xiang, N. , Wu, Y. , Li, C. , Chen, Q. , Li, D. , Liu, T. , Zhao, J. , Liu, M. , Tu, W. , Chen, C. , Jin, L. , Yang, R. , Wang, Q. , Zhou, S. , Wang, R. , Liu, H. , Luo, Y. , Liu, Y. , Shao, G. , Li, H. , Tao, Z. , Yang, Y. , Deng, Z. , Liu, B. , Ma, Z. , Zhang, Y. , Shi, G. , Lam, T.T.Y. , Wu, J.T. , Gao, G.F. , Cowling, B.J. , Yang, B. , Leung, G.M. , and Feng, Z. (2020) Early transmission dynamics in Wuhan, China, of novel Coronavirus–infected pneumonia. New England Journal of Medicine, 382, 1199–1207. 31995857 10.1056/NEJMoa2001316 PMC7121484

[radm12471-bib-0057] Li, Z. and Daspit, J.J. (2016) Understanding family firm innovation heterogeneity: a typology of family governance and socioemotional wealth intentions. Journal of Family Business Management, 6(2), 103–121.

[radm12471-bib-0058] Linnenluecke, M.K. and McKnight, B. (2017) Community resilience to natural disaster: the role of disaster entrepreneurship. Journal of Enterprising Communities: People and Places in the Global Economy, 11(1), 166–185.

[radm12471-bib-0059] Mahdad, M. , De Marco, C.E. , Piccaluga, A. , and Di Minin, A. (2020) Harnessing adaptive capacity to close the pandora’s box of open innovation. Industry and Innovation, 27(3), 264–284.

[radm12471-bib-0060] Mazzelli, A. , De Massis, A. , Petruzzelli, A.M. , Del Giudice, M. , and Khan, Z. (2020) Behind ambidextrous search: the microfoundations of search in family and non‐family firms. Long Range Planning, 53(6), 101882.

[radm12471-bib-0061] Miller, D. and Le Breton‐Miller, I. (2006) Family governance and firm performance: agency, stewardship, and capabilities. Family Business Review, 19(1), 73–87.

[radm12471-bib-0062] Miller, D. , and Le Breton‐Miller, I. (2014) Deconstructing socioemotional wealth. Entrepreneurship Theory and Practice, 38(4), 713–720.

[radm12471-bib-0063] Newey, L.R. and Zahra, S.A. (2009) The evolving firm: how dynamic and operating capabilities interact to enable entrepreneurship. British Journal of Management, 20(S1), S81–S100.

[radm12471-bib-0064] Nordqvist, M. , Sharma, P. , and Chirico, F. (2014) Family firm heterogeneity and governance: a configuration approach. Journal of Small Business Management, 52(2), 192–209.

[radm12471-bib-0065] Salvato, C. , Chirico, F. , and Sharma, P. (2010) A farewell to the business: championing exit and continuity in entrepreneurial family firms. Entrepreneurship & Regional Development, 22(3–4), 321–348.

[radm12471-bib-0066] Shepherd, D.A. and Zacharakis, A. (2000) Structuring family business succession: an analysis of the future leaders in decision making. Entrepreneurship Theory and Practice, 24(4), 25–39.

[radm12471-bib-0067] Simón‐Moya, V. , Revuelto‐Taboada, L. , and Ribeiro‐Soriano, D. (2016) Influence of economic crisis on new SME survival: reality or fiction? Entrepreneurship & Regional Development, 28(1–2), 157–176.

[radm12471-bib-0068] Smallbone, D. , Deakins, D. , Battisti, M. , and Kitching, J. (2012) Small business responses to a major economic downturn: empirical perspectives from New Zealand and the United Kingdom. International Small Business Journal, 30(7), 754–777.

[radm12471-bib-0069] Soluk, J. and Kammerlander, N. (2021) Digital transformation in family‐owned Mittelstand firms: a dynamic capabilities perspective. European Journal of Information Systems, 1–36. 10.1080/0960085X.2020.1857666

[radm12471-bib-0070] Soluk, J. , Miroshnychenko, I. , Kammerlander, N. , and De Massis, A. (2021) Family influence and digital business model innovation: the enabling role of dynamic capabilities. Entrepreneurship Theory and Practice, 104225872199894. 10.1177/1042258721998946

[radm12471-bib-0071] Stafford, K. , Danes, S.M. , and Haynes, G.W. (2013) Long‐term family firm survival and growth considering owning family adaptive capacity and federal disaster assistance receipt. Journal of Family Business Strategy, 4(3), 188–200.

[radm12471-bib-0072] Streeck, H. , Schulte, B. , Kuemmerer, B. , Richter, E. , Höller, T. , Fuhrmann, C. , Bartok, E. , Dolscheid, R. , Berger, M. , Wessendorf, L. , and Eschbach‐Bludau, M. (2020). Infection fatality rate of SARS‐CoV‐2 infection in a German community with a super‐spreading event. Accessed 2 May 2020 from https://www.ukbonn.de/C12582D3002FD21D/vwLookupDownloads/Streeck_et_al_Infection_fatality_rate_of_SARS_CoV_2_infection2.pdf/%24FILE/Streeck_et_al_Infection_fatality_rate_of_SARS_CoV_2_infection2.pdf 10.1038/s41467-020-19509-y PMC7672059 33203887

[radm12471-bib-0073] Tversky, A. and Kahneman, D. (1974) Judgment under uncertainty: heuristics and biases. Science, 185(4157), 1124–1131. 17835457 10.1126/science.185.4157.1124

[radm12471-bib-0074] Zahra, S.A. , Hayton, J. , and Salvato, C. (2004) Entrepreneurship in family vs. non‐family firms: a resource‐based analysis of the effect of organizational culture. Entrepreneurship Theory and Practice, 28(4), 363–381.

[radm12471-bib-0075] Zellweger, T.M. , Nason, R.S. , and Nordqvist, M. (2012) From longevity of firms to transgenerational entrepreneurship of families: introducing family entrepreneurial orientation. Family Business Review, 25(2), 136–155.

[radm12471-bib-0076] Zhu, N. , Zhang, D. , Wang, W. , Li, X. , Yang, B. , Song, J. , Zhao, X. , Huang, B. , Shi, W. , Lu, R. , and Niu, P. (2020) A novel Coronavirus from patients with pneumonia in China. New England Journal of Medicine, 382, 727–733. 31978945 10.1056/NEJMoa2001017 PMC7092803

